# EHMTI-0185. “Perceived stress index among school adolescents with primary headaches (tension-type headache or migraine) in Tashkent”

**DOI:** 10.1186/1129-2377-15-S1-B8

**Published:** 2014-09-18

**Authors:** S Gazieva, ANNA Prokhorova, N Rashidova

**Affiliations:** 1 Neurology, Tashkent Medical Academy, Tashkent, Uzbekistan

## Introduction

Psychosocial stress is one of the most important factors causing migraine and T-TH. Stressors of school environment are expected to be a probable cause of headaches.

The aim of the study is to investigate the susceptibility to stress among adolescents with T-TH and migraine.

## Material and methods

A total of 135 students with T-TH including 94 girls and 41 boys, 96 students with migraine including 70 girls and 26 boys, 52 students with migraine and coexisting T-TH including 38 girls and 14 boys, and 240 headache free students including 121 girls and 119 boys, aged 12-17 years, participated in the study. Stress influence was established according to the Rahe Stress Scale of perceived stress.

## Results

The average perceived stress index in the group with T-TH was 27.9%; 30.6% for girls and 25.1% for boys, in the group with migraine - 19.1%; 20.6% for girls and 17.6% for boys, with migraine and coexisting T-TH 29.5%; 33.5% for girls and 25.5% for boys. In headache free patients it was 17.9%; 18.4% for girls and 17.5% for boys. The perceived stress index revealed an increase with age of patients, frequency of T-TH and number of migraine days.

## Conclusions

Increased vulnerability to stress is present among adolescents with T-TH and migraine. The correlation between increased stress level and age, gender and frequency of headache was found.

No conflict of interest.

